# Predicting circulating biomarker response and its impact on the survival of advanced melanoma patients treated with adjuvant therapy

**DOI:** 10.1038/s41598-020-63441-6

**Published:** 2020-05-04

**Authors:** Itziar Irurzun-Arana, Eduardo Asín-Prieto, Salvador Martín-Algarra, Iñaki F. Trocóniz

**Affiliations:** 1 Pharmacometrics & Systems Pharmacology group, Department of Pharmacy and Pharmaceutical Technology, School of Pharmacy and Nutrition, University of Navarra, Pamplona, Navarra Spain; 2 IdiSNA, Navarra Institute for Health Research, Pamplona, Navarra Spain; 3 Department of Medical Oncology, Clínica Universidad de Navarra, Pamplona, Navarra Spain

**Keywords:** Melanoma, Tumour biomarkers

## Abstract

Advanced melanoma remains a disease with poor prognosis. Several serologic markers have been investigated to help monitoring and prognostication, but to date only lactate dehydrogenase (LDH) has been validated as a standard prognostic factor biomarker for this disease by the American Joint Committee on Cancer. In this work, we built a semi-mechanistic model to explore the relationship between the time course of several circulating biomarkers and overall or progression free survival in advanced melanoma patients treated with adjuvant high-dose interferon-${\boldsymbol{\alpha }}{\bf{2}}{\bf{b}}$. Additionally, due to the adverse interferon tolerability, a semi-mechanistic model describing the side effects of the treatment in the absolute neutrophil counts is proposed in order to simultaneously analyze the benefits and toxic effects of this treatment. The results of our analysis suggest that the relative change from baseline of LDH was the most significant predictor of the overall survival of the patients. Unfortunately, there was no significant difference in the proportion of patients with elevated serum biomarkers between the patients who recurred and those who remained free of disease. Still, we believe that the modelling framework presented in this work of circulating biomarkers and adverse effects could constitute an additional strategy for disease monitoring in advance melanoma patients.

## Introduction

According to the American Cancer Society, the incidence rate of melanoma has been rising for the last 30 years. Although the disease accounts for only about 1% of skin cancers, it is responsible for the death of the vast majority of these patients making it the most aggressive neoplasm of the skin^1^.

Since 1995, immunotherapy based on interferon-$\alpha$ containing regimens has been used as an adjuvant therapy to surgery for patients diagnosed of American Joint Committee on Cancer (AJCC) stage IIB, IIC or III melanoma after the Eastern Cooperative Oncology Group (ECOG) 1684 trial showed that a high-dose regimen of Interferon $\alpha$-2b (IFN-$\alpha 2b$) led to a significant prolongation of progression-free survival and overall survival (PFS and OS, respectively) compared to the control group^2^. Although new therapeutic strategies are emerging for advanced melanoma in recent years thanks to the FDA approval of several new immunotherapy and targeted drugs, treatment with IFN-$\alpha 2b$ still constitutes one of the alternatives in the therapeutic arsenal in many hospitals and health care centers. However, due to the toxicity and the evidence that only a subgroup of patients can benefit from this treatment, acceptance of IFN-$\alpha 2b$ among physicians is limited.

In order to adequately treat melanoma patients, it is important to study those factors related to the prognosis and outcome of the disease. As reflected in recent studies, the most important prognostic factors that could predict the outcome of melanoma patients include the vertical tumor thickness known as Breslow’s index, the presence of ulceration, the mitotic rate, the location of distant metastases, as well as the levels of serum lactate dehydrogenase (LDH)^1^. Other serum biomarker levels that have been proposed as possible prognostic factors are the melanoma-inhibiting activity (MIA) and the calcium binding protein S100B^3^, but no consensus exists on their prognostic capability.

Proper assessment of the predictive capacity of biomarkers longitudinal data should be done in the context of mechanistic computational models linking them with clinical outcome. Biomarker trajectories are usually not linear and show great variability across individuals. Consequently, a non-linear mixed effects (NLME) modelling approach provides a valuable option to handle and model this type of dynamic behaviour. In NLME models, individual profiles are characterized by a common structural model with fixed population parameters and a statistical model with random effects to allow the parameters to vary within the patient population. In this work, longitudinal biomarker data has been described based on semi-mechanistic pharmacokinetic-pharmacodynamic (PKPD) type models and linked to the PFS and OS. Recent efforts have shown that this approach is feasible to identify robust markers that allow the selection of patients that could obtain a therapeutic benefit from the different anticancer treatments and to improve the prediction of their survival^4–6^.

Therefore, in this study we aim to establish a quantitative treatment-biomarker-survival modelling framework using nonlinear mixed effects PKPD modelling to link the survival of advanced melanoma patients with LDH, MIA and/or S100B protein kinetics following IFN-$\alpha 2b$ administration. In addition and taking into account the toxicity associated to IFN-$\alpha 2b$ administration, neutropenic effects were also described mechanistically^7^ in the current evaluation providing a highly valuable approach in which to evaluate possible predictors of clinical response while minimizing adverse effects.

## Methods

### Patient characteristics and data collection

In this retrospective study, data related to different biomarker levels and patient survival were obtained from the medical records of 48 patients diagnosed with advanced melanoma and treated in the University Clinic of Navarra (Pamplona, Spain). The Research Ethics Committee from the University of Navarra approved the study protocol and informed consent for study participation was obtained from all patients. The protocol was carried out in accordance with the Declaration of Helsinki (Seoul 2008 version) and local laws and regulations.

Adult patients with histologically documented AJCC stage IIB, IIC, or III primary cutaneous melanoma were included in the dataset. All the patients were treated with adjuvant high-dose IFN-$\alpha 2b$ between 2004 and 2013. The high-dose regimen followed the Kirkwood scheme^2^: intravenous administration of 20 MU/m2/day at the induction phase (5 days/week during 4 weeks) followed by subcutaneous injections of 10 MU/m2/day during the maintenance phase (3 days/week during 48 weeks). Blood samples for drug quantification and tumor assessment measurements during treatment were not available.

Table 1 summarizes physiopathological and demographic characteristics of the patients included in the study and Table 2 summarizes the main adverse events reported during IFN-$\alpha 2b$ therapy.

**Table 1:** Demographic characteristics and diagnostic values of the patients*.

Demographic Characteristics	Overall population (N = 48)
Gender (M/F)	25/23
Age at melanoma diagnosis (years)	50 [21–74]
Body weight at first IFN dose (kg)	73 [45–108]
Height (cm)	168.5 [148–188]
BSA (m^2^)	1.825 [1.37–2.31]
***Diagnosis values***	
Location of primary lesion	
Face	3
Trunk	15
Extremity (Upper/lower)	4/19
Other	4
NR	3
Type of melanoma	
Amelanotic melanoma	1
Superficial spreading melanoma	16
Acral lentiginous melanoma	2
Maligna melanoma	14
Nodular melanoma	15
Laterality (Right/Left/NR)	11/14/23
Local recurrence (Yes/No/NR)	05/02/41
Diagnostic - Pathological stage AJCC 2009b	
IB	6
IIA/IIB	1/6
III/IIIA/IIIB/IIIC	2/8/9/4
NR	12
First dose - Pathological stage AJCC 2009b	
IIB	4
III/IIIA/IIIB/IIIC	10/8/14/7
NR	5
SLNB	
Yes (Positive cases)	32 (21)
No	16
History of complete lymphadenectomy	
Yes (Positive cases)	44 (41)
No	3
NR	1
Breslow thickness (mm)	
<1	5
$\ge$ 1 to <2	10
$\ge$ 2 to <4	13
$\ge$ 4	12
Clark level	
II	2
III	12
IV	22
V	2
NR	10
Ulceration (Yes/No/NR)	8/22/18
Extracapsular extension (Yes/No/NR)	3/35/10
Satellite lesions (Yes/No/NR)	3/24/21
BRAF Mutation (Yes/No/NR)	08/08/32
ECOG performance status (before therapy)	
0	14
1	21
NR	13

M: male; F: female; BSA, Body Surface area; AJCC, American Joint Committee on Cancer; SLNB, Sentinel Lymph Node Biopsy; ECOG, Eastern Cooperative Oncology Group; NR: Not reported.

*Continuous variables are expressed as median [range] whereas categorical variables are expressed as number of cases.

**Table 2:** Main adverse events reported during IFN therapy.

Main adverse events*	Induction phase	Maintenance phase
Neutropenia	13	6
Thrombocytopenia	2	1
Increased transaminases	12	3
Hepatotoxicity	8	1
Fatigue	9	8
Osteoarticular pain	1	2
Influenza-like symptoms	2	3
Fever	3	1
Headache	4	−
Anorexia	1	5
Depression	3	4
Nausea	2	2

*Other adverse events reported were: dermal events (cellulitis, dermatitis, skin dryness and alopecia), neurological events (anxiety, somnolence, insomnia, dizziness, recurrent syncope), weight loss and hyperthyroidism.

Blood samples for measurement of LDH, MIA and protein S100B were collected from each patient before, meanwhile and after therapy. For MIA and S100B levels, observations corresponding to 9 and 10 patients of the database were not reported, respectively. A total of 954/383/405 LDH/MIA/S100B observations were included in the analysis, where each patient contributed a mean of 19/10/10 samples (range 1-57/1-31/1-33).

## Data analysis

A population joint sequential modelling approach was used for the development of the treatment-biomarker-survival framework^8^. First, the relationship between treatment and biomarkers dynamics was characterized, and then their predicted time profiles were used to characterize the hazard rates and subsequently PFS and OS. For the continuous (biomarker levels, and absolute neutrophils counts) and non-continuous (PFS, OS) response data the first-order conditional estimation method with interaction and the Laplacian estimation method were used, respectively, for parameter estimation in NONMEM 7.3.

The continuous data of the different biomarkers and the absolute neutrophil counts (ANC) were logarithmically transformed for the analysis. The developed models share a common architecture constituted by a structural model and a statistical component where (i) between-subject variability (BSV) was modeled exponentially and (ii) residual variability was described using a proportional or an additive error model on the log-transformed data corresponding to the biomarker and ANC levels respectively.

### Model selection

Model selection during model building included comparison of the objective function value which is approximately equal to minus twice the log(likelihood) (−2LL) and inspection of graphical diagnostics. For application of the −2LL ratio test in the case of comparing nested models, a significance level of P < 0.01 was used, corresponding to a decrease in −2LL of at least 6.63 when one extra parameter was added. Non-nested models were compared using the Akaike information criteria (AIC)^9^.

### Model evaluation

Evaluation was performed through simulation-based diagnostics by performing visual predictive checks (VPC)^10^. VPCs evaluate the model’s ability to describe the median tendency and variability in the observed data. To this end, the original dataset was simulated 1000 times by sampling new sets of individual parameters from the estimated population parameter distributions. Then, 95% prediction intervals were derived from the simulation results, and compared with the 5th, 50th and 95th percentiles of the observed data. The results of the VPCs can also be normalized by the typical population prediction, creating the so-called prediction-corrected VPCs.

Precision of parameter estimates was obtained from the analysis of 500 bootstrap datasets. Briefly, in a bootstrap analysis, the original dataset is replaced to produce another dataset of the same size but with a different combination of individuals. This re-sampled database is then used to re-estimate the population and variability parameters of the model. Lastly, median values and 95% confidence intervals of the re-estimated parameter distribution are calculated.

### Software and tools

Preprocessing of the data, additional simulation exercises and graphical and other statistical analyses, including predictive checks and bootstrap analyses, were performed with Perl-speaks-NONMEM (PsN) software^11^, Simulx (http://simulx.webpopix.org/), R version 3.4.3 (http://www.R-project.org/) and Rstudio version 1.1.456 (http://www.rtudio.com/).

#### Model for biomarker response

As above mentioned, NLME models were used to characterize the longitudinal LDH, MIA and S100B protein concentrations over time.

As no PK data of interferon therapy were available from the patients, a K-PD modelling approach^12^ was used to study the link between the interferon dosing rate and the biomarker dynamics. A preliminary exploratory analysis showed that the decrease in biomarker levels occurred with some delay after treatment administration that was handled incorporating a series of transit compartments. Transit of the pharmacodynamic signal elicited by interferon through the chain of compartments was characterized by the first order rate constant ${k}_{tr}$ defined as $(n+\mathrm{1)/}MTT$, where $n$ is the number of transit compartments and $MTT$ the mean transit time between compartments.

In the absence of treatment, an exponential tumor growth governed by a first-order proliferation rate constant (${k}_{prol}$) was defined in the following form:

1 $$\frac{dTA}{dt}={k}_{prol}\cdot TA$$

where TA (Tumor Activity) represents the unobserved tumor progression dynamics. IFN-$\alpha 2b$ therapy induced tumor shrinkage, and hence, the final equation for TA was expressed as a balance between tumor growth and drug-induced tumor death:

2 $$\frac{dTA}{dt}={k}_{prol}\cdot TA-{f}_{drug}\cdot TA$$

Different models for drug effects (${f}_{drug}$) were explored including linear, ${E}_{max}$ and sigmoidal models. The value of TA at diagnosis ($T{A}_{0}$) was arbitrarily set to 1.

Lastly a turn-over model assuming that the circulating levels of biomarkers are a function of (i) a synthesis process governed by TA and the first-order rate constant ${k}_{in}$, and (ii) an elimination process controlled by the first-order rate constant ${k}_{out}$ as shown in the expression below:

3 $$\frac{dBiomarke{r}_{j}}{dt}={k}_{i{n}_{j}}\cdot TA-{k}_{ou{t}_{j}}\cdot Biomarke{r}_{j}$$

where j represents each of the biomarkers (LDH, MIA and S100B). The initial condition for biomarker values was estimated as due to tumor progression steady-state condition did not hold.

Each biomarker’s longitudinal data were fitted separately using the model equations described above. Afterwards, the kinetics of the three biomarkers was combined in the same analysis to evaluate their contribution in the clinical outcome of the patients.

#### Models for progression-free survival and overall survival

PFS and OS were modeled as time to event response data using parametric survival analyses. Time frame was considered between diagnosis and (i) time at which the patient showed disease progression or died and (ii) last recorded time (right censored). Interval censored for the case of PFS response was not considered in this evaluation.

Different distributions (exponential, Weibull and Gompertz) were used to describe the hazard rate, $hz(t)$, which is defined as the instantaneous risk of dying/recurring at each time provided that the patient lives/is free of disease to that time, with the sole restriction of being no-negative. In contrast to the hazard function, the survival function indicates the probability that the event of interest has not yet occurred by time t (the patient is still alive or free of disease) and therefore if the hazard function is known, the survival probability is automatically determined as follows:

4 $$S(t)=exp(\,-\,{\int }_{0}^{t}hz(t))$$

where $-{\int }_{0}^{t}hz(t)$ represents the cumulative hazard.

Time-varying covariates, as the predicted time course of the biomarkers, were included in the model as modulators of $hz(t)$. Parameters describing $hz(t)$ has no associated BSV as each patient contributed with a single measurement.

The effect of the predicted dynamics for the three biomarkers on $hz(t)$ were tested alone or in combination to explore whether their absolute or relative change from baseline over time were predictive of OS/PFS. For the estimation of the parameters linking the survival and the biomarker model, the population parameters from the previously selected biomarker model were fixed and the corresponding observed levels were retained together with the PFS and OS data (PPP $\&$ D method^13^).

#### Model for neutropenic adverse effects

A semi-mechanistic model for myelosupression^7^ was used to characterize the dynamics of the absolute neutrophil circulating counts under IFN-$\alpha 2b$ therapy. Briefly, in this model neutrophil development is determined by different physiological processes: (i) a self-renew first-order process of the precursor cells (ii) a maturation chain comprising three transit compartments (iii) a homeostatic regulation that modulates the proliferation of the precursor cells as a function of the change of ANC relative to the value at baseline ($AN{C}_{0}$), and finally (iv) a first-order elimination of ANC. As said before, no interferon PK data were available, and therefore, a K-PD model^12^ was used to link the dosing rate to drug effects. The model structure is defined by the following set of ordinary differential equations:

5 $$\frac{dIFNa2b}{dt}=-\,{K}_{e}\cdot IFNa2b$$

6 $$\frac{dProl}{dt}={k}_{PROL}\cdot Prol\cdot \mathrm{(1}-{E}_{DRUG})\cdot {\left(\frac{AN{C}_{0}}{ANC}\right)}^{\gamma }-{k}_{TR}\cdot Prol$$

7 $$\frac{dTransit1}{dt}={k}_{TR}\cdot Prol-{k}_{TR}\cdot Transit1$$

8 $$\frac{dTransit2}{dt}={k}_{TR}\cdot Transit1-{k}_{TR}\cdot Transit2$$

9 $$\frac{dTransit3}{dt}={k}_{TR}\cdot Transit2-{k}_{TR}\cdot Transit3$$

10 $$\frac{dANC}{dt}={k}_{TR}\cdot Transit3-{k}_{circ}\cdot ANC$$

where ${K}_{e}$ represents the first-order elimination rate constant of interferon after administration, ${k}_{PROL}$ is the first-order rate of proliferation on precursor cells (Prol), ${k}_{TR}$ is the first order rate constant governing the transit of immature neutrophils between transit compartments, ${k}_{circ}$, is the first-order rate constant of elimination of ANC and $\gamma$ is the parameter modulating the feedback mechanism. The transit rate was defined as ${k}_{TR}=(n+\mathrm{1)/}MT{T}_{ANC}$ where $MT{T}_{ANC}$ is the mean maturation time and $n$ is the number of transit compartments, which was three in this model. As no information was gathered from the precursor and immature cells, it was assumed that, at baseline, their number of cells were equal to $AN{C}_{0}$, and therefore the parameter values for ${k}_{PROL}$, ${k}_{TR}$ and ${k}_{circ}$ were defined to be equal. Both a linear and a sigmoidal ${E}_{max}$ function of the predicted levels of IFN-$\alpha 2b$ were evaluated for drug effects (${E}_{DRUG}$), which were assumed to act by reducing the proliferation rate of the neutrophils.

In order to reduce the number of parameters to estimate and improve model stability, the parameters reported in the original work^7^ for $MTT$ and $\gamma$ were used, as the authors demonstrated that the estimates of the system related parameters showed consistency across different anti-cancer agents. The final parameters to be estimated were reduced to $AN{C}_{0}$, parameters measuring drug effects and those quantifying random effects.

#### Covariate selection

Covariate model selection was performed using the Stepwise Covariate Model-building (SCM) tool in PsN^14^, which consists on a forward covariate inclusion followed by a backward deletion approach. Specifically, this technique consist on creating a full model by combining the covariates identified as significant (p < 0.05) and once the full model is established, each potential covariate is individually removed to see if the value of -2LL significantly increases (p < 0.01).

Patient characteristics are listed in Table 1. For those covariates that were correlated between them, as it was the case for weight, height and body surface area (BSA), only the most relevant covariate with regard to usual dose adjustments in the clinic, in this case BSA, was included in the analysis. Therefore, the following patient’s characteristics measured at baseline were explored for inclusion in the model (the covariates were tested in all the model parameters): Breslow thickness, presence of ulceration (yes vs. no), age, body surface area, type of melanoma (horizontal growth phase vs vertical growth phase) and ECOG performance status. Other a priori important clinical covariates like the presence of BRAF mutation or the mitotic rate were not studied as the number of missing data was high. The categorical level of invasion known as the Clark index was neither included in the analysis as almost every patient had reported a level of IV.

Covariates were tested for significance following the general model:

11 $$TVP={\theta }_{n}\cdot \mathop{\prod }\limits_{1}^{m}g(co{v}_{m},co{v}_{m,ref},{\theta }_{m})\cdot \mathop{\prod }\limits_{1}^{p}(1+\mathop{\sum }\limits_{cat\mathrm{=2}}^{ctg}{\theta }_{p,cat})$$

where the typical value of a parameter (TVP) was described as a function of $m$ continuous ($co{v}_{m}$) and $p$ categorical covariates (cat) with a total number categories of $ctg$. ${\theta }_{n}$ describes the ${n}^{th}$ typical parameter value for an individual with covariate values equal to the reference values: [$(co{v}_{m}=co{v}_{m,ref})$ and $cat$ = 1] where $co{v}_{m,ref}$ refers to the median value across the studied population. $g$ refers to the different linear and non-linear functions explored for the relationship between the values of $co{v}_{m}$ and $co{v}_{m,ref}$, and ${\theta }_{m}$ and ${\theta }_{p,cat}$ are parameters quantifying the magnitude of the covariate-parameter relationship.

## Results

A total of 30 (62 $\%$) and 21 (43 $\%$) patients completed the induction and the maintenance phase, respectively. In total, 17 patients (35 $\%$) had at least one dose reduction during the induction or maintenance phase due to adverse events and 25 patients (52 $\%$) had dose delays for the same reason, demonstrating the high toxicity of IFN-$\alpha 2b$ therapy.

### Exploratory analysis

**Figure 1:**
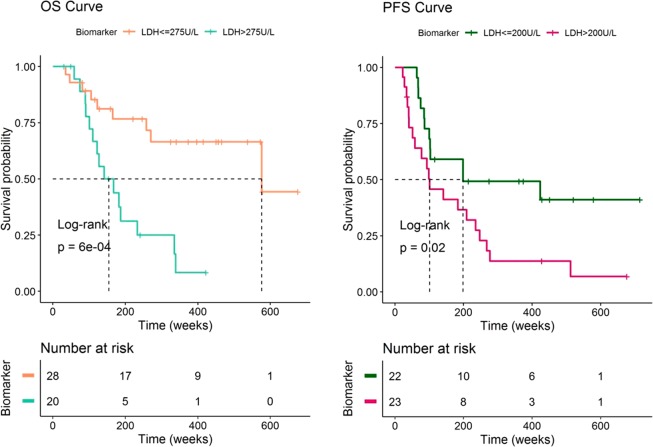
Evaluation of the overall survival (OS) and progression-free survival (PFS) of the patients with high and low biomarker concentrations at the end of the study. MIA and S100 biomarker Kaplan Meier curves showed equivalent results (see supplementary figures).

The median overall survival of the patients in the dataset was 270 weeks. A first exploratory analysis of the dataset showed that patients with high LDH, MIA and S100B levels at the end of the study have the poorest outcomes as indicated by the Kaplan–Meier curves of OS shown in Fig. 1 and Supplementary Figure S1. However, the Kaplan-Meier analysis and log-rank tests corresponding to the PFS response did not show significant results (p > 0.01) when stratifying by high and low biomarker values at the time of disease progression (Fig. 1 and Supplementary Figure S1 bottom). Other in principle relevant clinical covariates like the Breslow thickness, presence of ulceration, tumor extension (distal, localized or regional) or the value of the biomarkers before treatment initiation also showed no significant differences in OS or PFS (p > 0.01) (Supplementary Figures S2 and S3 respectively). These findings suggest that a link might exist between biomarker dynamics during and after IFN-$\alpha 2b$ treatment and OS and not for PFS.

**Figure 2:**
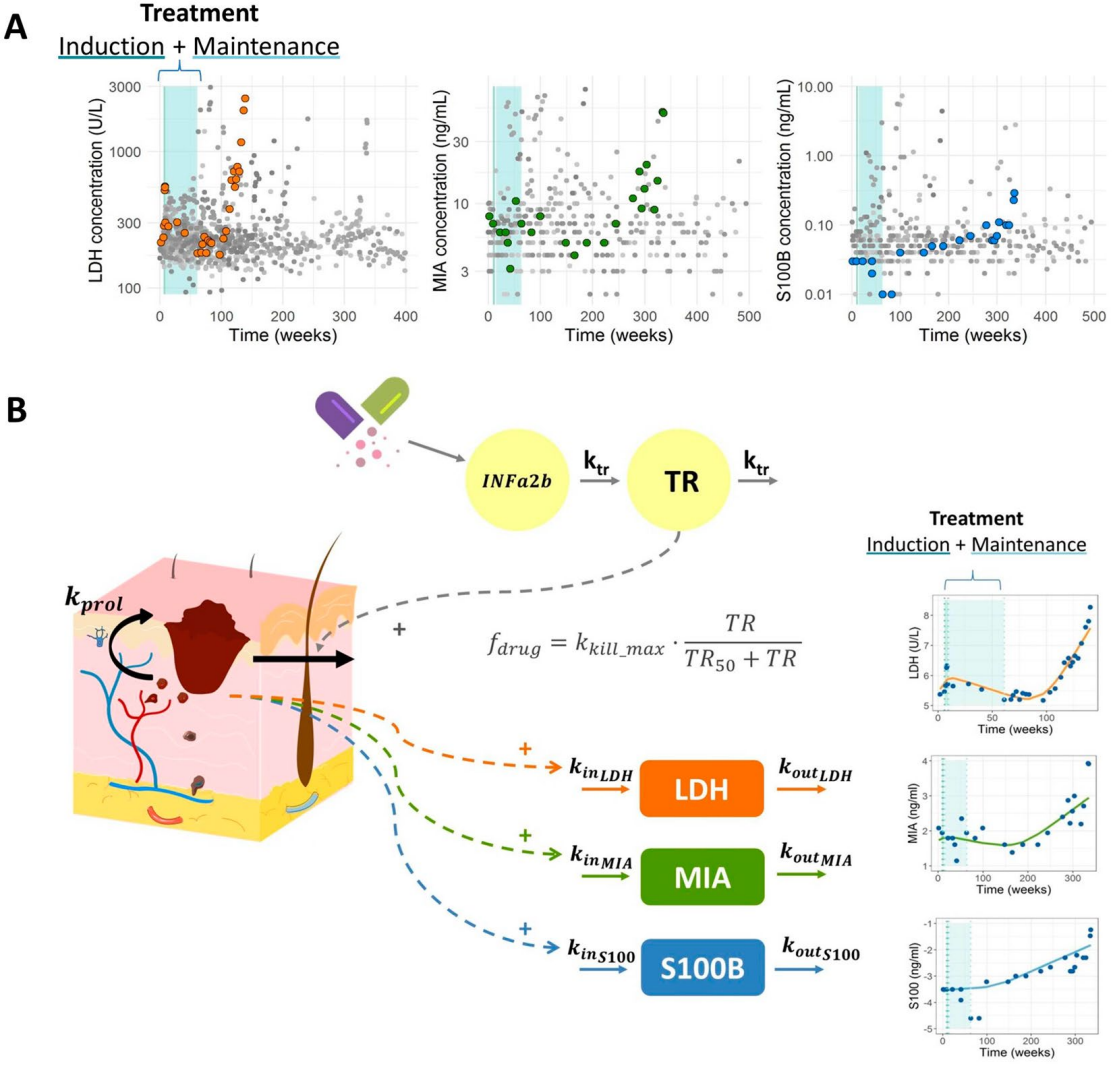
(**A**) Raw values (solid circles) of the different biomarker levels over time where the profile of one individual has been highlighted in color. The treatment period (induction phase followed by the maintenance phase) is shadowed in blue. (**B**) Schematic representation of the K-PD model proposed for the IFN-$\alpha 2b$ effect on LDH, MIA and S100 levels (left) and three individual biomarker profiles (right) where solid circles represent biomarker observation values and solid lines indicate the prediction of the model. Parameter abbreviations: ${k}_{prol}$, first-order tumor proliferation rate; MTT, mean transit time; ${A}_{50}$, amount of drug producing 50% of the maximum elimination; ${k}_{kill\_max}$, first-order tumor elimination rate; ${k}_{in}$ first-order biomarker synthesis rate constant; ${k}_{out}$, first-order degradation rate constant.

The raw values for each biomarker are shown in Fig. 2A, where the time course for one individual data and its treatment period (induction phase followed by the maintenance phase) has been highlighted. When looking at the whole range of observations, it is difficult to observe a general trend in the data. However, when the biomarker profiles are observed individually, a response to the therapy followed by a relapse after the treatment period can be detected. In this work, we intended to describe this trend and its link to the OS and PFS data using semi-mechanistic computational models.

### Biomarker dynamics

Figure 2B provides a schematic representation of the model which is described by the following set of ordinary differential and algebraic equations:

12 $$\frac{dIFNa2b}{dt}=-\,{k}_{tr}\cdot IFNa2b$$

13 $$\frac{dTR}{dt}={k}_{tr}\cdot IFNa2b-{k}_{tr}\cdot TR$$

14 $$\frac{dTA}{dt}={k}_{prol}\cdot TA-{f}_{drug}\cdot TA$$

15 $${f}_{drug}={k}_{kill\_max}\cdot \frac{TR}{TR+T{R}_{50}}$$

16 $$\frac{dBiomarke{r}_{j}}{dt}={k}_{i{n}_{j}}\cdot TA-{k}_{ou{t}_{j}}\cdot Biomarke{r}_{j}$$

The transit compartments included in the model to describe the delayed response to IFN-$\alpha 2b$ might be reflecting the processes involved in the activation of the drug-related immune-modulatory response. Other models reflecting different hypothesis, for example including delays in the tumor activity or in the biomarker dynamics were also considered, but their performance (evaluated as −2LL value) was worse in comparison with the model finally selected. Drug effects were described with an ${E}_{max}$ model were $T{R}_{50}$ is the predicted pharmacodynamic signal generated by the treatment in the transit compartment eliciting half of maximum effect (${k}_{kill\_max}$). The rest of parameter abbreviations have been defined in *Methods*.

Parameter estimates and their corresponding BSV are summarized in Table 3. For the sake of parameter identifiability, the value of $T{R}_{50}$ was fixed in the model of LDH and MIA dynamics after performing a sensitivity analysis study (data not shown). For S100B tumor marker the population estimate and BSV of MTT were also fixed to the values obtained in the model for LDH concentrations. None of the studied covariates had a significant effect on the model parameters.

**Table 3:** Final model parameter estimates.

	Typical estimate	BSV: CV%
***Model for biomarker response***		
	LDH	
MTT (weeks)	22.3 (12.49–37.318)	59.8 (44.7–113.5)
$T{R}_{50}(U\cdot {10}^{6})$	23.7 (-)	—
LDH baseline (U/L)	225 (167.082–261.393)	31.3 (21.45–51.28)
${k}_{prol}(week{s}^{-1})$	0.0029 (0.00196–0.0053)	58 (51.75–224.27)
${k}_{kill\_max}(week{s}^{-1})$	0.0077 (0.0056–0.012)	34 (31.3–128.45)
${k}_{out}(week{s}^{-1})$	0.321 (0.189–0.822)	—
Residual error (%)	0.0521 (0.0376–0.0576)	NA*
	MIA	
MTT (weeks)	33.1 (28.04–36.9)	63.8 (41.6–83)
$T{R}_{50}(U\cdot {10}^{6})$	25.2 (-)	—
MIA baseline (ng/mL)	7.53 (5.57–7.85)	45.3 (27–54.8)
${k}_{prol}(week{s}^{-1})$	0.0028 (0.0022–0.0042)	75.6 (69.2–141)
${k}_{kill\_max}(week{s}^{-1})$	0.0058 (0.003–0.0061)	34.6 (25.5–62.4)
${k}_{out}(week{s}^{-1})$	0.369 (0.288–0.486)	—
Residual error (%)	0.248 (0.192–0.296)	NA
	S100B	
MTT (weeks)	22.3 (-)	59.8 (-)
$T{R}_{50}(U\cdot {10}^{6})$	19.9 (16.42–23.64)	—
S100B baseline (ng/mL)	0.0503 (0.038–0.0656)	42.4 (36.74–55.95)
${k}_{prol}(week{s}^{-1})$	0.0023 (0.0017–0.0025)	56.2 (45.8–73.48)
${k}_{kill\_max}(week{s}^{-1})$	0.0065 (0.005–0.0072)	—
${k}_{out}(week{s}^{-1})$	1.99 (1.71–2.49)	—
Residual error (%)	0.348 (0.3–0.424)	NA
***OS model***		
$\lambda$	0.00181 (0.0016–0.0026)	—
${\beta }_{\Delta LDHrel}$	1.1 (0.59–1.9)	—
***Myelosuppresion model***		
$MT{T}_{ANC}(weeks)$	0.52	—
$AN{C}_{0}{(10}^{9}/L)$	3.41 (3.134–3.737)	32.4 (26.3–40)
$Slope(U\cdot {10}^{-4})$	0.0425 (0.0392–0.055)	—
${K}_{e}(week{s}^{-1})$	0.389 (0.344–0.566)	52.4 (24.36–70.26)
$\gamma$	0.161	—
Residual error (${10}^{9}/L$)	0.485 (0.465–0.501)	—

^*^NA: Not Applicable.

90% confidence intervals (in parenthesis) were obtained from 500 bootstrap analyses.

Estimates of between-subject variability (BSV) are shown as coefficients of variation.

Parameter names are defined in the text.

**Figure 3:**
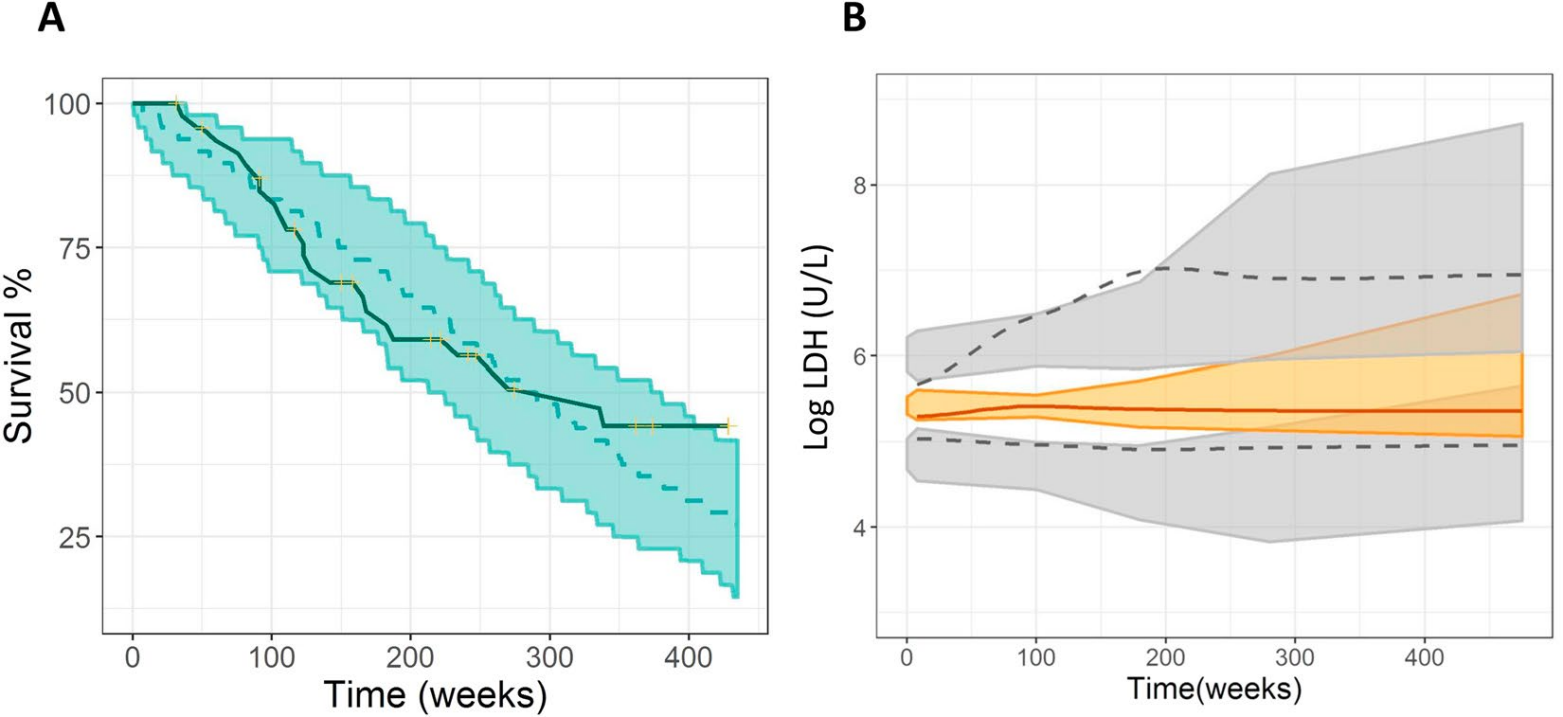
Model evaluation: Visual Predictive Checks. (**A**) Kaplan Meier plot of OS probability. The solid blue line represents raw data while the blue shaded area cover the 95% prediction interval calculated from 1000 simulated studies. (**B**) VPC of the selected biomarker model. Median (solid line), ${5}^{th}$ and ${95}^{th}$ percentiles (dashed lines) of the observed data. 95% confidence Intervals for median (shaded colored area), ${5}^{th}$ and ${95}^{th}$ percentiles (shaded grey areas) of the simulated data.

The analysis of the Individual Weighted Residuals (IWRES) vs. time or predicted biomarker values is shown in Supplementary Figure S4. Additionally, the three individual fits of a representative patient for each biomarker in Fig. 2 and the results of the VPCs represented in Fig. 3A demonstrated good agreement between observed and simulated data (only the VPC for LDH is shown).

With respect to parameter precision, none of the 95% confidence intervals for the model parameters reported in Table 3 (computed from the bootstrap analysis) included the value of zero, indicating that the data supported the degree of complexity of the final model selected. In all the models, ${k}_{prol}$ and MTT showed a high BSV value and a wide range for the confidence interval of the BSV.

### Survival model

Predicted biomarker dynamics over time were linked to the probability of survival as an argument of the baseline hazard function, which was best described using an exponential model with constant $\lambda$ in the case of OS and with a Gompertz function in the case of PFS (Supplementary Figure S5). Relative change from baseline of LDH ($\Delta LDHrel$) was the most significant predictor of OS (p < 0.001), however none of the biomarker dynamics significantly improved PFS predictions as previously suggested by the Kaplan-Meier curves from Fig. 1 and Supplementary Figure S1. Additionally, none of the studied covariates (see *Methods* for the information about the covariates tested in the model) influenced survival according to the univariate analysis done in PsN using the SCM tool and therefore none of them were included in the joint model afterwards.

The final survival model for has the following form:

17 $$hz(t)=\lambda \cdot {e}^{\beta \cdot \Delta LDHrel(t)}$$

where the term ${e}^{\beta \cdot \Delta LDHrel(t)}$ describes the change in $hz$ elicited by the relative change from baseline of LDH for each individual $i$ multiplied by the link parameter $\beta$. The estimated values for $\alpha$ and $\beta$ are summarized in Table 3 and the corresponding VPC for OS is shown in Fig. 3B. We only considered time up to 450 weeks after diagnosis to evaluate model performance through VPCs as for longer times only 7 individuals were remaining for a period of approximately 225 weeks more.

The predicted median 2-year and 5-year overall survival probability computed was 84.37% and 58.33% respectively, which were very similar to the observed values of 82.4% and 56.39% obtained from the 48 patients in our dataset. Additional simulation exercises where the therapy was administered in the same time period to all the individuals showed that a 50% decrease in tumor proliferation practically did not affect the 2-year survival rate, but increased the 5-year and 10-year rate a 13.7% and 42% respectively (see Supplementary Figure S6).

### Model for neutropenic adverse effects

**Figure 4:**
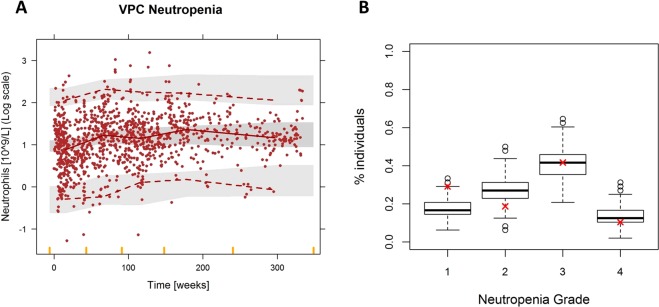
Evaluation of the myelosuppression model for the absolute neutrophil counts (ANC) of the patients. (**A**) Prediction-corrected visual predictive check. Solid circles represent observed ANC, solid lines represent the median of the observed data, and dashed lines the 2.5 and 97.5 percentiles of the observations. Shaded areas are the 95% confidence intervals based on the simulated data (n = 1000) for the corresponding percentiles. (**B**) Percentage of patient in grade 1,2,3 and 4 neutropenia (grade 1: >1.5 ANC, grade 2: 1–1.5 ANC, grade 3: 0.5–1 ANC, grade 4: <0.5 ANC). Boxplots summarize the result of the 500 simulations and the red cross represents the real percentage values from the dataset.

Table 2 summarizes the main adverse events reported during interferon therapy. Due to the fact that neutropenia was one of the most reported and potential life threatening toxic effects, we decided to characterize this adverse response using the semi-mechanistic model from^7^. That semi-mechanistic myelosuppression model adequately described the time course of the log-transformed absolute neutrophil counts as illustrated by the prediction-corrected VPC from Fig. 4A. The linear drug effect model showed significantly better fitting results compared with the ${E}_{max}$ model (p < 0.01). The final model included BSV in the ANC baseline parameter ($AN{C}_{0}$) and in the elimination rate constant (${K}_{e}$) of the K-PD model (see Table 3 for parameter values). None of the studied covariates had a significant effect on the model parameters.

In Fig. 4B the percentage of patients with grade 1, 2, 3 and 4 neutropenia calculated from five hundred simulated ANC vs time profiles were compared to the corresponding percentages derived from the observations. Results show that the model captures well severe grades of neutropenia.

## Discussion

A joint model for the dynamics of circulating biomarkers and overall survival has been established and evaluated in patients with melanoma during treatment with IFN-$\alpha 2b$. Additionally, a myelosuppression model was also developed to evaluate the adverse effects of the IFN-$\alpha 2b$ therapy in the same cohort of patients. This framework enables to convert the individual biomarker levels into personalized predictions of survival while taking toxicity into account. All of the investigated biomarkers were significantly related to OS when evaluated one by one, but the relative change from baseline of LDH was identified as the most predictive of OS regarding objective function values. Although other studies also showed a significant association between LDH and PFS in melanoma^15^, in our analysis none of the tumor marker dynamics significantly improved PFS predictions. Moreover, treatment with Interferon is more associated with an improvement in PFS rather than OS but our data did not allow us to characterize this link.

**Figure 5:**
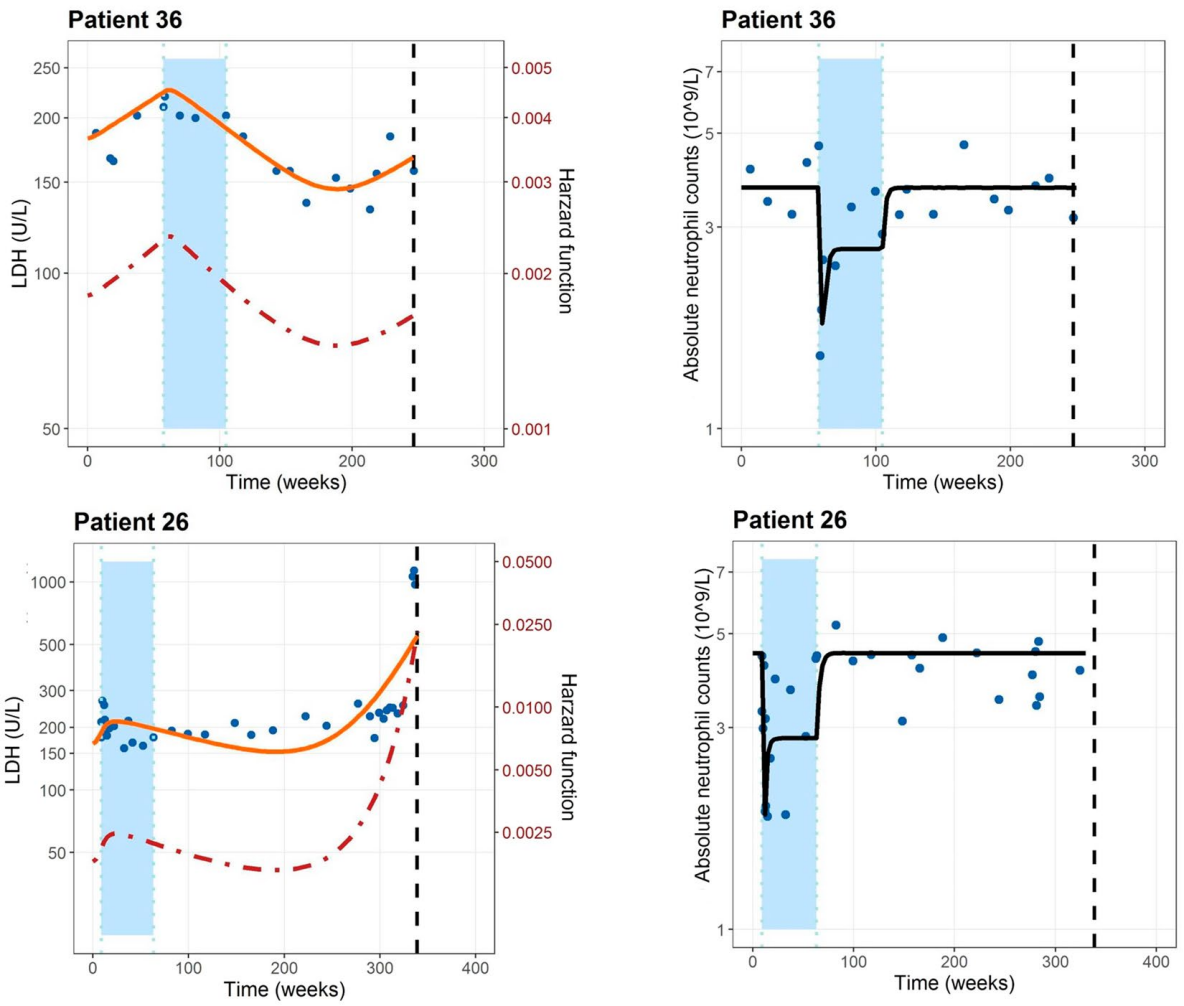
Individual predicted LDH and ANC profiles (solid lines) by the selected models and the time course of the hazard rate (dashed line) differentiating by an individual who is alive at the last follow-up (patient 36) and an individual who died (patient 26). Solid points represents the observation values of the patients.

Serum LDH, which is a standardized biomarker routinely monitored in clinic, is also used to categorize patients with stage IV melanoma, as increased LDH values are known to be correlated with a poor outcome of the patients. However, the link between biomarker values and survival needs to be quantitatively characterized in order to allow for more meaningful predictions of patient prognosis. In this work, we add insights in this context by providing a treatment-biomarker-survival-toxicity framework where the effectiveness of alternate dosing regimens could be tested based on $\Delta LDHrel$ values and neutropenia. Figure 5 conceptualizes the computational framework as it shows the individual LDH and ANC profiles and the time course of the hazard rate differentiating by an individual who is alive at the last follow-up (patient 36) and an individual who died (patient 26). In this figure it can also be appreciated that the effect of the therapy on the ANC was much faster than the decrease in LDH. This justifies the differences found between the estimates for the ${k}_{tr}$ parameter which had a value of 0.039 weeks-1 for the case of LDH and the ${K}_{e}$ of the myelosuppression K-PD model which had a value of 0.389 weeks^−1^ (almost 10 times higher).

The treatment of advanced stage melanoma has evolved immensely in recent years with the success of new immunotherapies and targeted drugs^16^. Nowadays, it is well known that approximately 60% of melanomas harbor a mutation in the gene encoding for the serine/threonine protein kinase BRAF, which leaded to the development of selective BRAF inhibitors such as vemurafenib and dabrafenib. Although it has been demonstrated that these targeted drugs significantly improve PFS and OS in comparison with chemotherapy, the patients receiving this treatment rapidly develop resistance^16^. On the other hand, the FDA-approved checkpoint inhibitors against cytotoxic T lymphocyte antigen 4 (CTLA-4) and programmed death 1 (PD-1) enhance the natural antitumor immune response of the patients and also lead to improved survival^17^. However, only a subset of patients respond to immune checkpoint inhibitors and resistance mechanisms can also arise among this group of responders. In this context, the identification of predictive biomarkers and/or baseline covariates able to select patients most likely to benefit from these therapies could be crucial.

In our case, none of the studied baseline covariates (Breslow thickness, tumor extension, presence of ulceration…) influenced survival. Unexpectedly, this agrees with the result of other statistical analysis made in advanced melanoma patient data where the univariate analysis of the gender, age, Breslow thickness, BRAF mutation status and location of primary tumor resulted in no significant association with OS^18,19^. Still, we find difficult to conclude that these covariates do not influence the survival of melanoma patients because we think that part of the results obtained were influenced by the small number of patients in the dataset and the missing information regarding the covariates of these subjects. Therefore, a better univariate and multivariate baseline covariate analysis is encouraged if more informative datasets are available in the future. Other limitations to highlight regarding this work were that no PK and tumor progression measurements were available for the development of the model. That is why a K-PD approach was used to link the dosing records with a drug effect in an unobserved variable that simulates the disease progression of the patients. This tumor progression was in turn linked to the time course of LDH, MIA and S100B serum concentration dynamics that were produced by a first-order rate constant and cleared at a first-order elimination rate in healthy subjects.

However, the major obstacle to develop this treatment-biomarker-survival-toxicity framework to monitor clinical response in melanoma was the moderate efficacy of IFN-$\alpha 2b$ therapy. Although the 1684 ECOG trial probed a significant improvement on the PFS and OS of melanoma patients, subsequent trials showed limited efficacy of this treatment as monotherapy, particularly on the OS of the individuals^20^. Supplementary Table S1 shows how doubling the dose from the induction or the maintenance phase of the treatment influenced the LDH values, OS and ANC of 1000 simulated individuals for a 1, 2, 5 and 10 year period. The values summarized in this table showed that doubling the dose of the induction or maintenance phases doesn’t have much repercussion in OS due to the low drug effects, but altering the maintenance phase could provoke a lower neutropenia grade. That is the reason why interferon therapy is no longer the standard of care in high risk, resected melanoma in most of the developed countries. Nonetheless, the use of IFN-$\alpha 2b$ as a plausible option for patients with stage IIB/IIC melanoma and ulcerated primary tumor, and for patients with stage II and III melanoma with ulcerated primary tumor in countries with no access to new drugs, has not been ruled out as indicated recently by Spagnolo *et al*.^21^. In addition, the development of new treatments opens the opportunity to reanalyze the utility of these tumor markers as prognostic factors (in fact, LDH has been reported as a clinically significant factor associated with OS under targeted and immune therapies^18,19^) and to follow-up patients during therapy^22^ re-using parts of the model built in this work. More importantly, the modelling effort developed here offers an attractive methodology to evaluate not only new treatment alternatives in drug development but also existing ones in the clinic, in order to evaluate safety and efficacy of the therapy, identify predictive factors and biomarkers and finally, perform dosing optimization in order to improve the clinical outcome of the patients.

## Supplementary information

Supplementary information.

Supplementary codes.

## Data Availability

As NONMEM 7.3 is not an open-source platform, we provide the codes to simulate all the models described in this work with the R package Simulx in the Supplementary Material. Any additional dataset or code are available from the corresponding author on reasonable request.

## References

[1] Garbe C (2016). Diagnosis and treatment of melanoma. european consensus-based interdisciplinary guideline - update 2016. Eur. J. Cancer.

[2] Kirkwood JM (1996). Interferon alfa-2b adjuvant therapy of high-risk resected cutaneous melanoma: the eastern cooperative oncology group trial EST 1684. J. Clin. Oncol..

[3] Palmer SR, Erickson LA, Ichetovkin I, Knauer DJ, Markovic SN (2011). Circulating serologic and molecular biomarkers in malignant melanoma. Mayo Clin. Proc..

[4] Buil-Bruna N (2014). A population pharmacodynamic model for lactate dehydrogenase and neuron specific enolase to predict tumor progression in small cell lung cancer patients. AAPS J..

[5] Tang M (2016). Myeloma cell dynamics in response to treatment supports a model of hierarchical differentiation and clonal evolution. Clin. Cancer Res..

[6] Desmée S, Mentré F, Veyrat-Follet C, Sébastien B, Guedj J (2017). Using the SAEM algorithm for mechanistic joint models characterizing the relationship between nonlinear PSA kinetics and survival in prostate cancer patients. Biometrics.

[7] Friberg LE, Henningsson A, Maas H, Nguyen L, Karlsson MO (2002). Model of chemotherapy-induced myelosuppression with parameter consistency across drugs. J. Clin. Oncol..

[8] Desmée S, Mentré F, Veyrat-Follet C, Guedj J (2015). Nonlinear mixed-effect models for prostate-specific antigen kinetics and link with survival in the context of metastatic prostate cancer: A comparison by simulation of two-stage and joint approaches. AAPS J..

[9] Akaike, H. Factor analysis and AIC. In *Selected Papers of Hirotugu Akaike*, 371–386 (Springer, 1987).

[10] Bergstrand M, Hooker AC, Wallin JE, Karlsson MO (2011). Prediction-corrected visual predictive checks for diagnosing nonlinear mixed-effects models. AAPS J..

[11] Lindbom L, Ribbing J, Jonsson EN (2004). Perl-speaks-NONMEM (PsN)–a perl module for NONMEM related programming. Comput. Methods Programs Biomed..

[12] Jacqmin P (2007). Modelling response time profiles in the absence of drug concentrations: Definition and performance evaluation of the K–PD model. J. Pharmacokinet. Pharmacodyn..

[13] Zhang L, Beal SL, Sheiner LB (2003). Simultaneous vs. sequential analysis for population PK/PD data i: best-case performance. J. Pharmacokinet. Pharmacodyn..

[14] Lindbom L, Pihlgren P, Jonsson N (2005). PsN-Toolkit—A collection of computer intensive statistical methods for non-linear mixed effect modeling using NONMEM. Comput. Methods Programs Biomed..

[15] Gray MR (2014). Metastatic melanoma: lactate dehydrogenase levels and CT imaging findings of tumor devascularization allow accurate prediction of survival in patients treated with bevacizumab. Radiology.

[16] Azijli K, Stelloo E, Peters GJ, Van Den Eertwegh AJM (2014). New developments in the treatment of metastatic melanoma: immune checkpoint inhibitors and targeted therapies. Anticancer Res..

[17] Rausch, M. P. & Hastings, K. T. Immune checkpoint inhibitors in the treatment of melanoma: From basic science to clinical application. In Ward, W. H. & Farma, J. M. (eds.) *Cutaneous Melanoma: Etiology and Therapy* (Codon Publications, Brisbane (AU), (2018). 29461774

[18] Kelderman S (2014). Lactate dehydrogenase as a selection criterion for ipilimumab treatment in metastatic melanoma. Cancer Immunol. Immunother..

[19] Long GV (2016). Factors predictive of response, disease progression, and overall survival after dabrafenib and trametinib combination treatment: a pooled analysis of individual patient data from randomised trials. Lancet Oncol..

[20] Kirkwood JM (2000). High- and low-dose interferon alfa-2b in high-risk melanoma: first analysis of intergroup trial E1690/S9111/C9190. J. Clin. Oncol..

[21] Spagnolo, F., Boutros, A., Tanda, E. & Queirolo, P. The adjuvant treatment revolution for high-risk melanoma patients. *Semin. Cancer Biol*. (2019). 10.1016/j.semcancer.2019.08.024 31445219

[22] Sanmamed MF (2014). Relevance of MIA and S100 serum tumor markers to monitor BRAF inhibitor therapy in metastatic melanoma patients. Clin. Chim. Acta.

