# Supplementary material for: Predicting circulating biomarker response and its impact on the survival of advanced melanoma patients treated with adjuvant therapy
Source: Sci Rep. 2020 May 4;10:7478. doi: 10.1038/s41598-020-63441-6 (PMC7198615; doi:10.1038/s41598-020-63441-6)
Supplement: Supplementary file 1 — Supplementary information. [file 41598_2020_63441_MOESM1_ESM.pdf]

Predicting circulating biomarker response and its impact on the survival of advanced melanoma patients treated with adjuvant therapy

Itziar Irurzun-Arana[1,2], Eduardo Asín-Prieto[1,2], Salvador Martín-Algarra[2,3], and Iñaki F. Trocóniz[1,2,*]

[1]Pharmacometrics & Systems Pharmacology group, Department of Pharmacy and Pharmaceutical Technology, School of Pharmacy and Nutrition, University of Navarra, Pamplona, Navarra, Spain.
[2]IdiSNA, Navarra Institute for Health Research, Pamplona, Navarra, Spain.
[3]Department of Medical Oncology, Clínica Universidad de Navarra, Pamplona, Navarra, Spain.

Supplementary Tables

Table S1. Median value (5–95th) of different outcomes obtained after 1000 simulation of different dosing schedules.

Standard dosing regimen*: induction: 20 MU/m^2 5 days a week + maintenance: 10 MU/m^2 3 days a week				
	1-year	2-year	5-year	10-year
LDHrel	-0.127 (-0.29-0.095)	-0.242 (-0.52-.019)	-0.062 (-0.61-1)	0.138 (-0.6-1.79)
Overall Survival	91.5 (89.7-93.2)	83.6 (81.3-85.9)	58.5 (55.5-61.6)	29.5 (26.8-32.4)
Absolut neutrophil count ($10^9/L$)	2.14 (1.04-4.07)	3.21 (1.94-5.36)	3.17 (1.93-5.66)	3.92 (2.37-6.15)
Regimen 2*: induction: 40 MU/m^2 5 days a week + maintenance: 10 MU/m^2 3 days a week				
LDHrel	-0.133 (-0.3-0.088)	-0.247 (-0.53-.018)	-0.07 (-0.62-0.99)	0.129 (-0.619-1.74)
Overall Survival	91.6 (89.9-93.3)	83.7 (81.4-86)	58.8 (55.8-61.9)	29.7 (27-32.7)
Absolut neutrophil count ($10^9/L$)	2.14 (1.03-4.07)	3.21 (1.94-5.36)	3.17 (1.93-5.66)	3.92 (2.36-6.14)
Regimen 3*: induction: 20 MU/m^2 5 days a week + maintenance: 20 MU/m^2 3 days a week				
LDHrel	-0.132 (-0.3-0.09)	-0.266 (-0.53-.016)	-0.1 (-0.66-0.94)	0.134 (-0.66-1.62)
Overall Survival	91.5 (89.8-93.2)	83.6 (81.3-86)	59.8 (56.8-62.9)	31.5 (28.7-34.5)
Absolut neutrophil count ($10^9/L$)	1.4 (0.4-3.27)	3.21 (1.93-5.36)	3.15 (1.93-5.66)	3.87 (2.3-6.1)

*The treatment started 6 weeks after diagnosis for all the regimens simulated. The treatment was administered to an individual with a body surface area of 1.8 m^2. LDHrel is the relative change of LDH from the baseline value.

Supplementary Figures

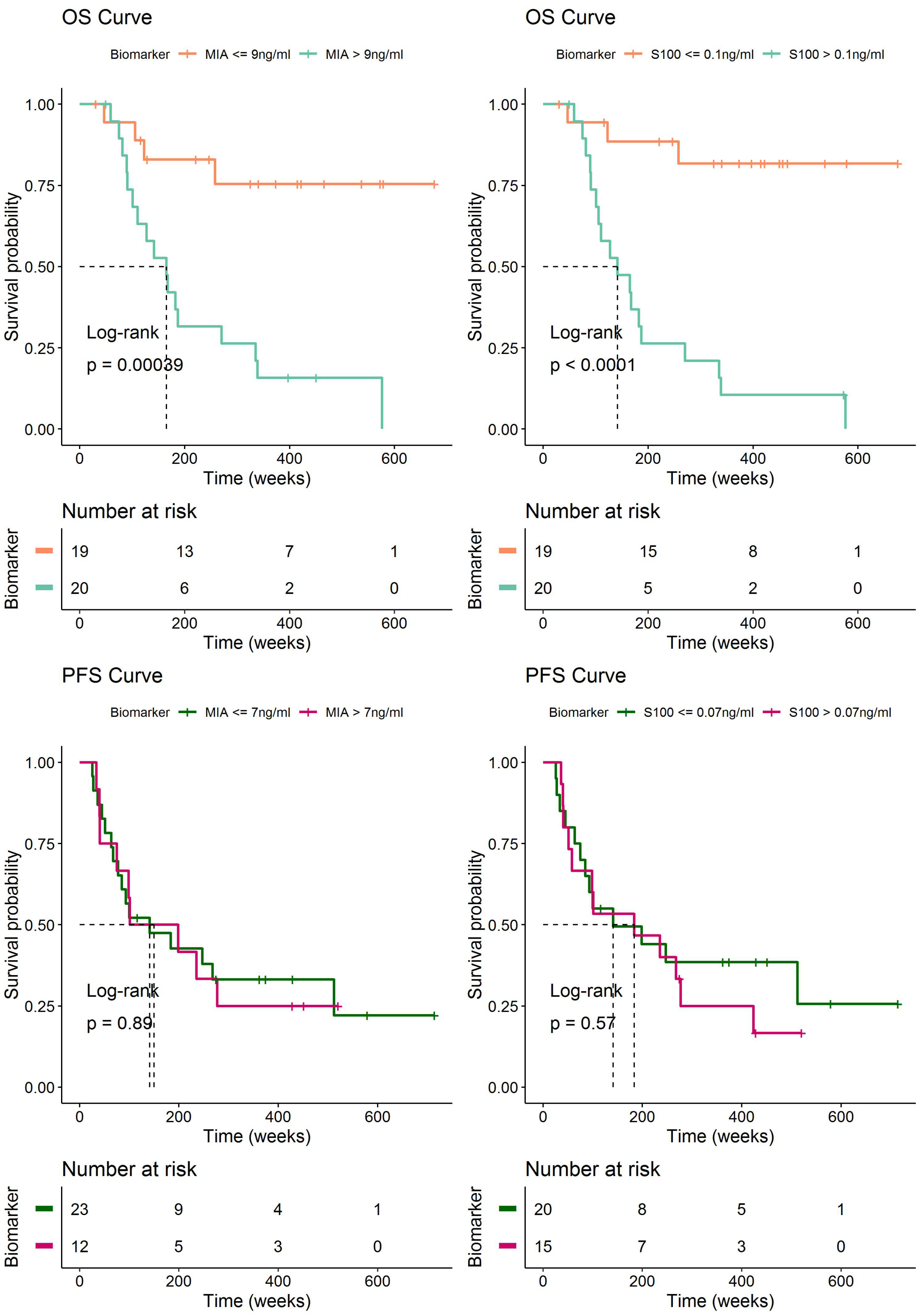

Figure S1. Evaluation of the overall survival (OS) and progression-free survival (PFS) of the patients with high and low MIA and S100B concentrations at the end of the study.

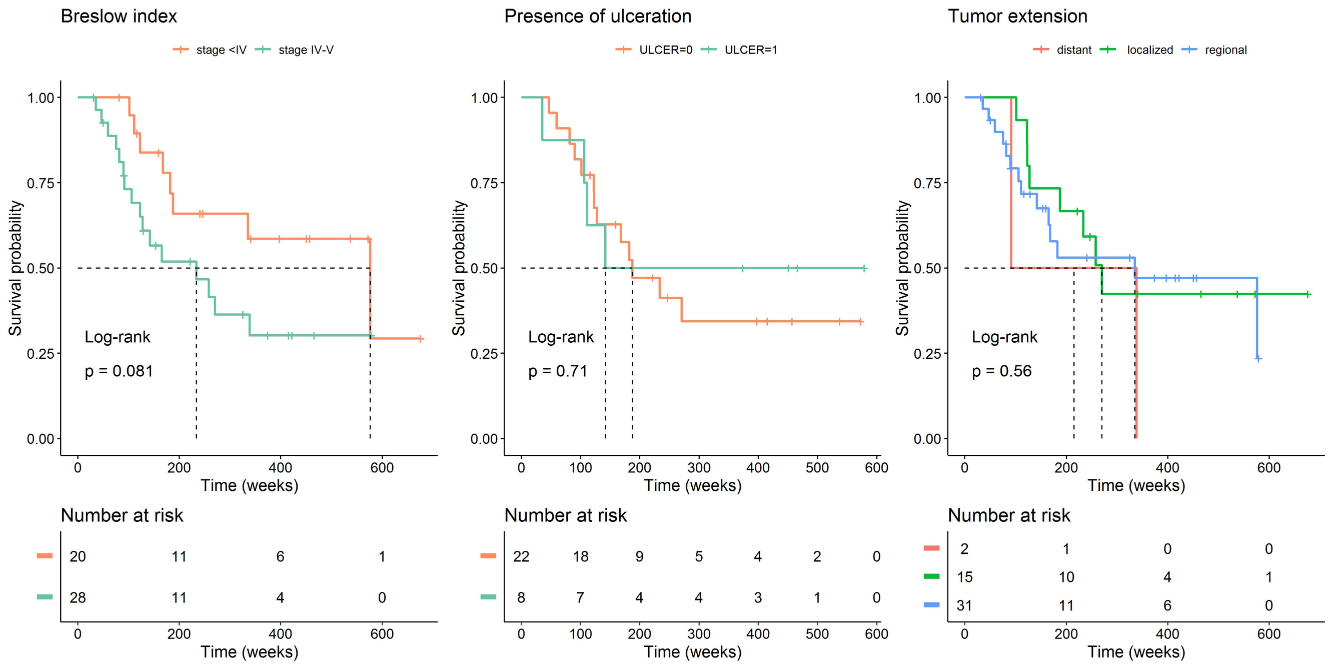

Figure S2. Kaplan-Meier curves of the overall survival stratified by different baseline covariates: Breslow index, the presence of ulceration and the tumor extension.

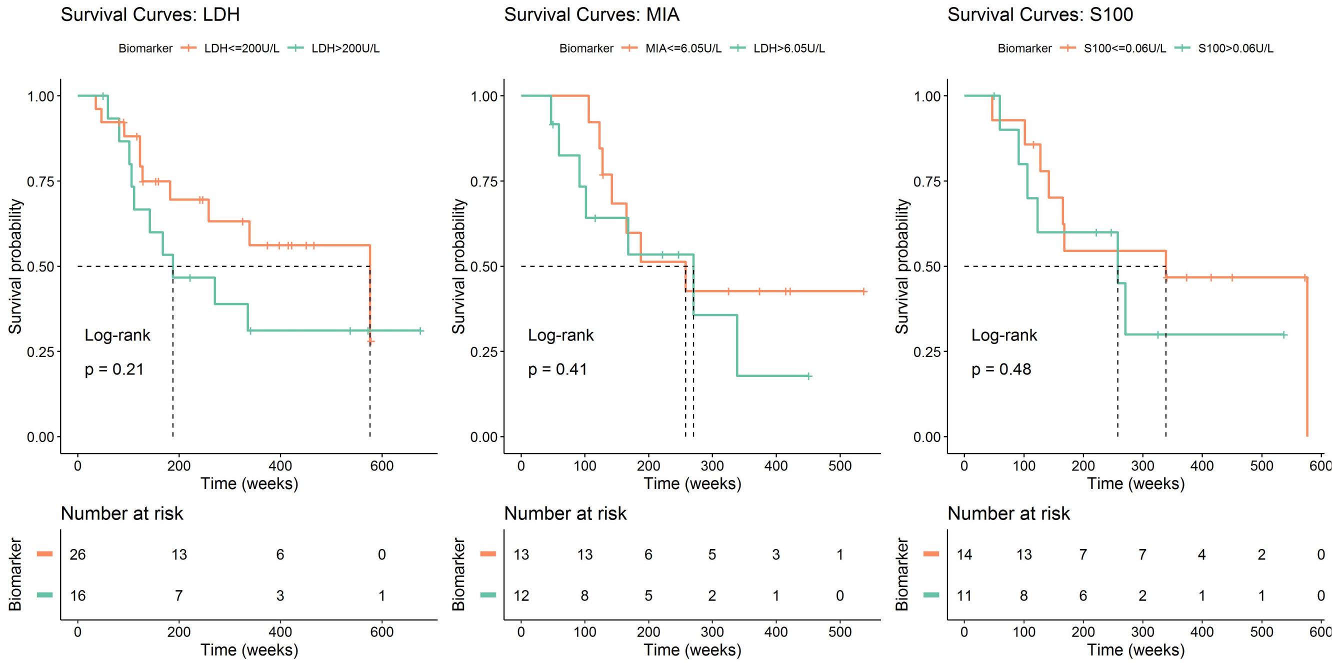

Figure S3. Kaplan-Meier curves of the overall survival stratified by baseline biomarker values (before treatment initiation).

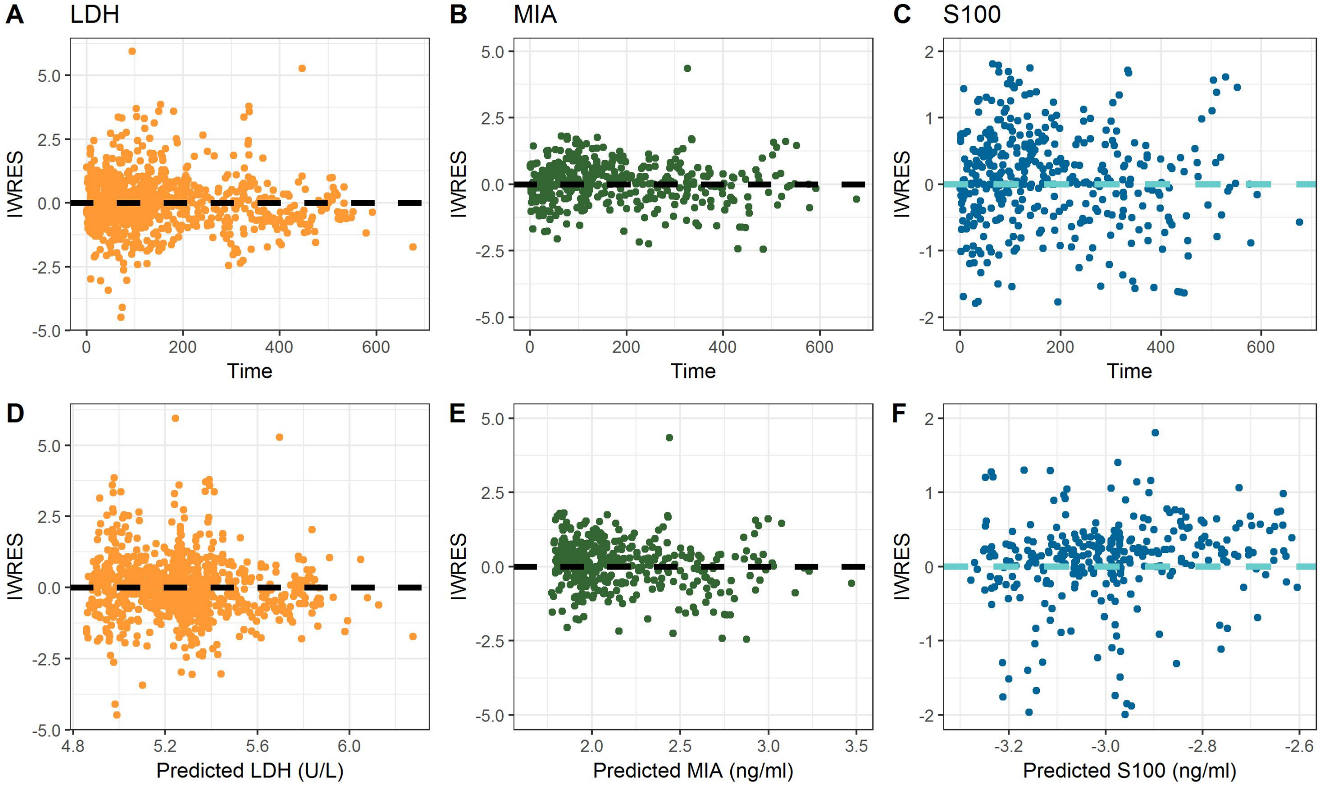

Figure S4. Residual error model diagnostics:Individual Weigthed RESiduals (IWRES) over time and vs model predictions.

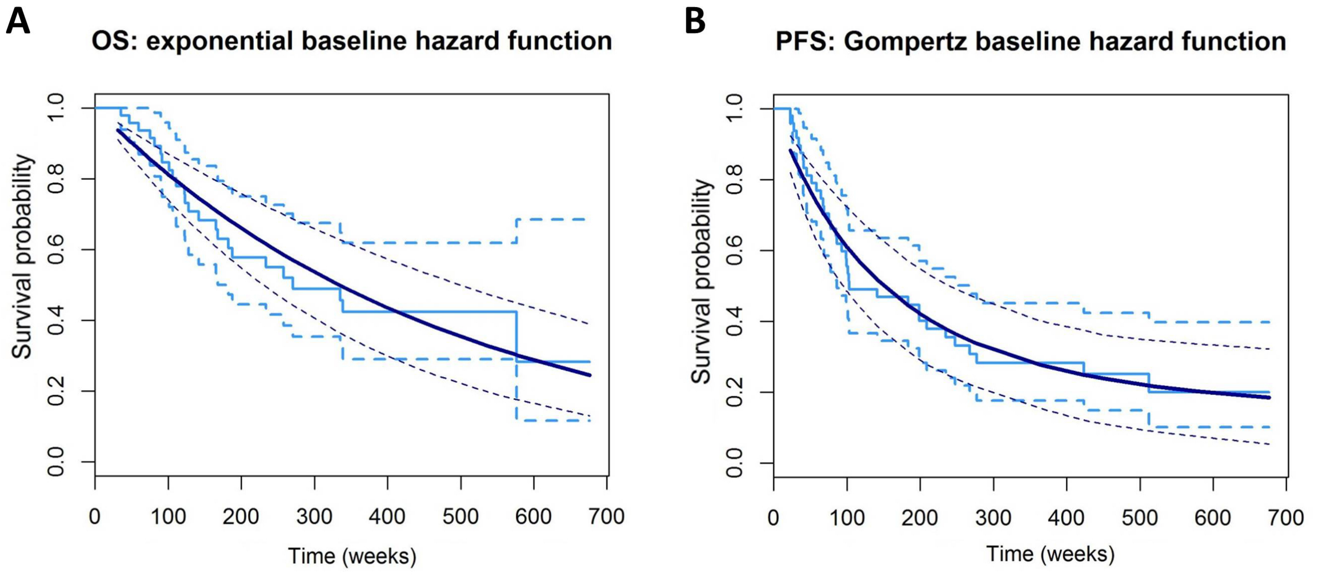

Figure S5. Best fitted models for the baseline hazard function of the overall survival (OS) and progression-free survival (PFS) of the patients. Light blue lines represent raw data and dark blue lines indicate the output of the model (the solid lines represent the median tendency and the dashed lines represent he 95% confidence interval)

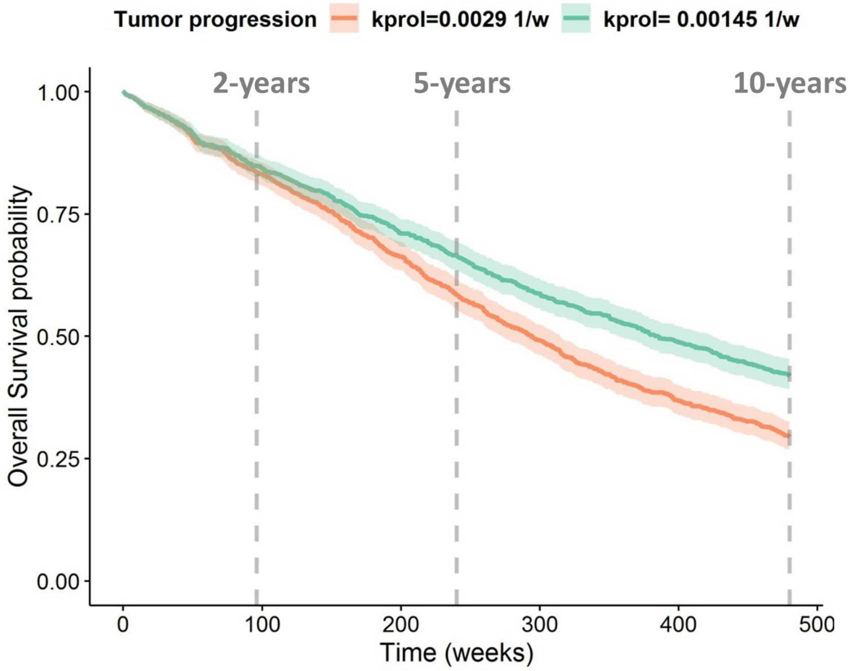

Figure S6. Kaplan Meier overall survival stratified by different values of the tumor proliferation rate constant parameter (k_{prol}). The plot is the result of the simulation of 1000 individuals receiving the treatment in the same time period.
